# Prevention of venous thromboembolism in patients undergoing major orthopedic surgery in China: a qualitative study of patients’ perceptions

**DOI:** 10.1186/s13018-018-0813-2

**Published:** 2018-04-24

**Authors:** Yaping Xu, Jing Zhao, Yuzhao Chen

**Affiliations:** 1 Department of Bone and Joint Surgery, China-Japan Friendship Hospital, 2 Yinghua Dongjie, Hepingli, Chaoyang District, Beijing, 100029 China; 2 Department of Trauma Orthopedics, China-Japan Friendship Hospital, Beijing, China; 3 Bone Necrosis and Joint Preservation Reconstruction Center, China-Japan Friendship Hospital, Beijing, China; 4 Department of Nursing, China-Japan Friendship Hospital, Beijing, China

**Keywords:** Thematic analysis, Qualitative descriptive, Education, Patient adherence, Quality of care, Postoperative complications

## Abstract

**Background:**

Venous thromboembolism (VTE) is the third leading cause of cardiovascular-associated death worldwide, and VTE prevention is one of the top patient safety strategies that hospitals can adopt. This study aimed to understand patients’ perceptions of VTE prevention related to major orthopedic surgery in order to guide the clinical practice of medical staff and improve patient quality of life. Patients undergoing major orthopedic surgery should receive interventions to prevent VTE. To encourage patient participation, these interventions should be patient-centered. However, few studies have examined the perceptions of VTE prevention among patients undergoing major orthopedic surgery.

**Methods:**

Participants were purposively selected from among patients undergoing major orthopedic surgery in the orthopedic department of a level-three, class-A hospital in Beijing, China. Data were collected through in-depth semi-structured interviews, and findings were based on a thematic content analysis. All interviews were held during each patient’s hospital stay.

**Results:**

From eight patients who participated, the following themes were identified: (1) unclear understanding of VTE, (2) poor understanding of the severity of postoperative VTE, and (3) poor understanding of VTE prevention.

**Conclusions:**

There are weak links in clinical care related to VTE prevention. We should aim to more completely understand patients’ needs, strengthen the health education provided to patients, and improve patient adherence to preventative measures.

## Background

Venous thromboembolism (VTE) is the third leading cause of cardiovascular-associated death worldwide [1], and hospital-acquired VTE is an important patient safety issue. The US Department of Health and Human Services’ agency for Healthcare Research and Quality (AHRQ) considers VTE prevention to be one of the top patient safety strategies that hospitals can employ [2–5]. Despite published guidelines [6, 7], the prevention of VTE remains a particularly challenging for patients undergoing major orthopedic surgery, including total hip arthroplasty, total knee arthroplasty, and hip fracture surgery.

Patients undergoing major orthopedic surgery should receive thromboprophylactic interventions, including health education, properly implemented basic prevention strategies, physical prevention strategies, and appropriate antithrombotic drugs. Ideally, these interventions should be patient-centered in order to encourage patient participation and adherence. However, few studies have examined patient perceptions of VTE prevention in relation to major orthopedic surgery; therefore, this study aimed to explore this topic in order to more accurately guide the clinical practices of medical staff and to improve the quality of life of patients undergoing major orthopedic surgery.

VTE includes deep vein thrombosis (DVT) and pulmonary embolism (PE). According to Virchow’s triad [8], the pathogenesis of VTE includes three main factors: hypercoagulability, reduced blood flow or stasis, and vessel damage due to injury or disease. Patients undergoing major orthopedic surgery have all three risk factors and are at an extremely high risk of developing VTE [9–12]. VTE results in substantial morbidity and is an important cause of perioperative and unexpected in-hospital deaths [13]. In these patients, it is necessary to combine basic prevention, physical prevention, and appropriate antithrombotic drugs according to the guidelines [11–13]. Moreover, despite the use of thromboprophylaxis, VTE remains a significant complication among patients undergoing major orthopedic surgery [14]. Therefore, the prevention of VTE in these patients is particularly important. In this study, we interviewed patients undergoing major orthopedic surgery to explore their knowledge and understanding of VTE prevention. We followed up the participants over the telephone 35 days after the surgery; none of the patients showed any symptoms of VTE.

## Methods

### Study design

A descriptive qualitative design was used. Between March and July 2017, a purposive sample of eight participants was selected from among patients attending the orthopedic department of a level-three, class-A hospital in Beijing, China. The participants selected for this study met the following inclusion criteria: over 18 years of age, undergoing major orthopedic surgery for the first time, able to communicate normally, no medical history of VTE, and able to provide informed consent to participate in the study. Face-to-face, semi-structured interviews were held during each patient’s hospital stay.

Saturation is the point in the process of data collection at which no new or relevant data emerges [15]. Using this principle, the study team observed a repetition of themes starting in the eighth interview; therefore, no further participants were recruited for the study.

### Data collection

Participants were asked to describe their knowledge and understanding of VTE prevention in the context of major orthopedic surgery (Table 1). Their responses were collected during audio-recorded, semi-structured interviews. Prior to the interviews, we established a relationship of trust with each participant by explaining our research objectives and assuring that their anonymity would be maintained. The interviews were conducted in a quiet office without disturbances. The content of the interviews was adjusted according to the situation, and we maintained a neutral attitude during the interview in order to encourage the participants to fully express their views. Each participant was interviewed once or twice, for a period of 20–40 min each time. In addition to audio recording, field notes were taken to record non-linguistic data during the interview in order to ensure the integrity and authenticity of the information. The audio recordings were converted to text (verbatim) within 24 h after the interview. Once the participant approved the text version of the interview, the audio recordings were deleted.

**Table 1:** Semi-structured interview guide

1. Have you ever heard of venous thromboembolism? Please talk about your perceptions of venous thromboembolism.	
2. Do you think there is a need to prevent venous thromboembolism during surgery? Please explain what you know about such prevention measures.	
3. Do you think there is a need to prevent venous thromboembolism after discharge from the hospital? Please explain what you know about such prevention measures.	
4. Have you received any health education about the prevention of venous thromboembolism from medical staff?	
5. Would you prefer to receive health advice from a doctor or a nurse?	
6. Do you have any other perceptions of the prevention of venous thromboembolism?	

### Data analysis

In accordance with the theory of qualitative methodology, the data collection and analysis occurred simultaneously [16]. The preliminary findings were incorporated into the subsequent data collection activities and were explored in depth. A qualitative content analysis, aimed at finding manifest and latent meanings within the data, was applied. Two experienced qualitative investigators coded the written transcripts to identify emerging categories, themes, and patterns [17]. Throughout the analysis, memorandums were written about the categories and their properties. The investigators integrated the needs of each participant and provided a reasonable interpretation to reveal the relationships among themes [16].

## Results

The characteristics of the eight patients included in the study are shown in Table 2. The data were grouped into the following themes: (1) unclear understanding of VTE, (2) poor understanding of the severity of postoperative VTE, and (3) poor understanding of VTE prevention strategies.

**Table 2:** Participant characteristics (*n* = 8)

Characteristic	Category	Participants (*n*)
Age (years), mean (range)		65 (44–92)
Sex	Male	3
	Female	5
Employment status	Full-time	2
	Retired	4
	None	2
Educational level	Primary school	1
	Middle school	3
	High school	1
	Some college or college	3
Marital status	Married	7
	Widowed	1
Admitting diagnosis	Knee osteoarthritis	4
	Hip osteoarthritis	1
	Osteonecrosis of the femoral head	2
	Femoral neck fracture	1
Surgical procedure	Total knee arthroplasty	3
	Unicompartmental knee arthroplasty	1
	Total hip arthroplasty	4

### Understanding of VTE

All eight study participants had heard of VTE. Three participants talked specifically about DVTs and PE as follows: “A colleague had a broken leg, which was put in a cast at the hospital and then he went home. He didn’t know there was a DVT, and soon he died of pulmonary embolism” (Participant 1); “My sister suffered from serious varicose veins in her lower limbs. After having an operation, her doctor told us it was important to prevent DVT, which could lead to PE and death” (Participant 6); and “During hospitalization, the medical staff talked to me about VTE. I know VTE includes two types: DVT and PE. My surgery increases the risk of DVT, and DVT may cause PE; it’s a disaster” (Participant 8). The other five participants mistook cerebral thrombosis for VTE; they described the following: “I’ve heard of some elderly people with cerebral thrombosis” (Participant 2); “My father had a cerebral thrombosis” (Participant 4); “I have high blood pressure and coronary heart disease. I’m afraid that I might get a cerebral thrombosis” (Participant 5); and “The medical staff told me, but I don’t think it’s important, and now I’m not very sure what VTE is” (Participant 7).

### Understanding of the severity of postoperative VTE

When talking about perioperative VTE prevention, only two participants thought it to be important, stating: “My colleague died of VTE. Once the VTE happens, the consequences are serious, and I attach great importance to the prevention of VTE” (Participant 1) and “My mother had a cerebral thrombosis. Now she has a hemiplegia, and her life was seriously affected. I think it’s very necessary to do something to prevent it. What the medical staff said is good for me” (Participant 5). Nevertheless, the other six participants did not understand the severity of postoperative VTE. Some expressed their thoughts as follows: “My father had a cerebral thrombosis. It is not fatal and I don’t think it’s too dangerous” (Participant 4) and “I was nervous when my doctor talked about complications, but now I feel okay. My operation is not major surgery. As for whether to prevent VTE, I am not against the prevention of VTE. During the hospital stay, I followed the doctor’s advice. I feel it is less important after I leave the hospital” (Participant 8).

When talking about whether to prevent VTE after hospital discharge, three participants made it clear that this was less important than the prevention while in hospital. They said, “I’m recovering well now. There is no need to prevent [VTE] after discharge” (Participant 2) and “I’m getting more and more active, and won’t need much attention when I go home” (Participant 7). However, five participants believed that attention was necessary after discharge: “As for whether to prevent VTE after discharge, I would happily follow your advice” (Participant 1); “I’m well now. It is good to be more careful after discharge” (Participant 3); “The effect should be consolidated, but I don’t know how long this will last” (Participant 4); and “The doctor told me that I need to continue to prevent [VTE] after leaving the hospital. I believe that’s important for me and I’d like to follow the doctor’s advice” (Participant 6).

### Understanding of VTE prevention strategies

All participants recognized some VTE prevention strategies. For example, they indicated that “Anti-embolism stockings are too tight and uncomfortable. I often take them off. If you tell me what it does in advance, I’d love to wear [them]. When it comes to rivaroxaban, I know it’s an antithrombotic, and I don’t know about its side effects, but I follow my doctor’s advice” (Participant 1); “My charge nurse told me how to do ankle pump exercises to prevent VTE. I don’t quite understand how this works. She didn’t tell me what the principle was. But I believe it’s good for me” (Participant 4); and “I see other patients wear anti-embolism stockings, and I take it for granted. I take antithrombotic drugs on time. The doctor told me about its functions and precautions, but I forget. Following the doctor’s advice is always correct” (Participant 6).

## Discussion

Patients undergoing major orthopedic surgery are at an extremely high risk of developing VTE. The prevention of VTE requires the joint efforts of the management, medical staff, patients, and their caregivers. This study aimed to identify manifest and latent meanings within the data and tried to consider this important, but sensitive, complication from the patients’ point of view. An unclear understanding of VTE, the severity of postoperative VTE, and VTE prevention strategies must be taken seriously.

Our study showed that most participants had an unclear idea of what VTE is, a finding consistent with those of previous studies. For example, Najafzadeh et al. [18] reported that fewer than half of the study patients had a clear understanding of DVT and PE, whereas all had a basic understanding of stroke and myocardial infarction. Similarly, in our study, more than half of the participants mistook cerebral thrombosis for VTE. Lee et al. [19] also showed participants to have insufficient knowledge of VTE, including its symptoms, risk factors, and prevention strategies; the authors identified a need for greater patient and caregiver education about VTE prevention, risks, and treatment side effects. To some extent, nurses experience difficulty with delivering health education. In 2015, Tang et al. showed that nurses had a lower understanding of VTE [20], which could affect how they presented health education on this topic.

Postoperative VTE can seriously impact patient quality of life. Our study revealed that most participants had an inadequate understanding of the severity of postoperative VTE. This was a result of their lack of knowledge regarding the harm associated with VTE and because medical staff was not specifically allocated to provide health education. The survey by Popoola et al. [21] emphasized that patients wanted to learn, via a variety of methods, about the harms associated with VTE.

Regarding VTE prevention, once participants were made aware of the severity of VTE, they expressed their willingness to adopt relevant preventative measures. All participants in our study expressed confidence in their physician’s expertise. Although they had an unclear idea of what VTE is and a poor understanding of the severity of postoperative VTE, the participants nonetheless adhered to preventative measures—albeit to varying degrees—as prescribed by their physicians. In the study by Najafzadeh et al. [18], all patients stated that they used antithrombotic medications as prescribed by their physicians; most patients stated that their decision to use the prescribed medications was mainly based on their trust in the expertise of their physician. Nurses should provide the patients with relevant information about diseases to help them to follow their doctor’s advice. Efforts to improve VTE prophylaxis, thereby decreasing the preventable harm associated with VTE, should target the entire continuum of care and should involve a variety of stakeholders, including patients and their families [21].

This is, to our knowledge, the first qualitative study involving patients’ perceptions of VTE prevention related to major orthopedic surgery. The qualitative approach allowed us to explore dimensions that have not yet been covered by the literature. This study reflected on how patients understood DVT and provided preliminary important information to aid future research aimed at quantitatively measuring patient perceptions of VTE prevention.

Our study has several limitations. First, participants were purposively selected from patients undergoing major orthopedic surgery in the orthopedic department of a level-three, class-A hospital. Therefore, our results may not be generalizable to other types of practice and medical institution settings. Second, the small sample size may limit the generalizability of our results. Nonetheless, when we reviewed the eighth participant, no new or relevant data were noted. Third, all interviews were conducted post-surgery, and therefore, the possibility of the patients’ perception being influenced by the medical staff was not accounted for.

## Conclusions

In summary, examining patients’ perceptions about VTE prevention when undergoing major orthopedic surgery revealed weak links in the provision of clinical care. Undoubtedly, patient behavior is influenced by their level of knowledge. Hence, in clinical practice, we should strengthen health education initiatives and aim to improve patient adherence to treatment. In addition, nursing staff should improve their professional competence; in turn, managers should provide diversified, targeted, and specialized training for nursing staff.

## Abbreviations

AHRQ: Agency for Healthcare Research and Quality
DVT: Deep vein thrombosis
PE: Pulmonary embolism
VTE: Venous thromboembolism
